# A minimally invasive pre-operative measure of total pulmonary resistance is associated with early adverse outcomes following total cavopulmonary connection

**DOI:** 10.1093/ehjimp/qyag126

**Published:** 2026-07-14

**Authors:** Niromila Nadarajan, Vivek Muthurangu, Michael A Quail

**Affiliations:** Research Department of Children’s Cardiovascular Disease, Institute of Cardiovascular Science, University College London, London WC1N 1DZ, UK; Department of Paediatric Cardiology, Great Ormond Street Hospital for Children NHS Foundation Trust, Great Ormond Street, London WC1N 3JH, UK; Research Department of Children’s Cardiovascular Disease, Institute of Cardiovascular Science, University College London, London WC1N 1DZ, UK; Research Department of Children’s Cardiovascular Disease, Institute of Cardiovascular Science, University College London, London WC1N 1DZ, UK; Department of Paediatric Cardiology, Great Ormond Street Hospital for Children NHS Foundation Trust, Great Ormond Street, London WC1N 3JH, UK

**Keywords:** single ventricle congenital heart disease, total cavopulmonary connection, bidirectional cavopulmonary connection, total pulmonary resistance, early Fontan failure, cardiovascular magnetic resonance

## Abstract

**Aims:**

Survival following total cavopulmonary connection (TCPC) is influenced by pre-operative haemodynamics. We developed a minimally invasive method to measure total pulmonary resistance (TPR) at the bidirectional cavopulmonary connection (BCPC) stage, reflecting the combined pulmonary impedance of the TCPC circulation. We evaluated whether TPR predicted early Fontan failure [EFF; death, TCPC take-down, emergency fenestration, or mechanical circulatory support (MCS) ≤ 6months post-TCPC] or prolonged post-operative intensive care unit (ICU) stay (>14 days).

**Methods and results:**

TPR was derived using jugular venous pressure as a surrogate for central venous pressure (CVP) and pulmonary blood flow (*Q*_p_) measured non-invasively from cardiovascular magnetic resonance (CMR). Indexed TPR (TPRi) was calculated as CVP/*Q*_p_ indexed to body surface area. Logistic regression evaluated predictors of early post-TCPC outcomes. Receiver operating characteristic (ROC) analysis assessed TPRi discrimination for EFF or the composite endpoint of EFF or prolonged ICU stay [early adverse outcome (EAO)]. Of 232 patients with BCPC undergoing CMR, 96% (*n* = 223) proceeded to TCPC. TPR data were available in 204 patients. On multivariable analysis, TPRi (adjusted OR 1.28, *P* = 0.030) and isomerism [adjusted odds ratio (OR) 14.34, *P* = 0.002] were independently associated with EFF. Both TPRi (adjusted OR 1.37, *P* = 0.001) and isomerism (adjusted OR 14.53, *P* = 0.001) also independently predicted EAO. ROC analysis identified optimal TPRi thresholds of ≥5.78 WU·m^2^ for EFF (sensitivity 80%, specificity 54%) and ≥5.7 WU·m^2^ for EAO (sensitivity 75%, specificity 53%).

**Conclusion:**

TPRi, calculated by combining pressure and flow data at the BCPC stage, independently predicts early adverse post-TCPC outcomes and may help identify high-risk patients without cardiac catheterization.

## Graphical Abstract

**Graphical Abstract:**
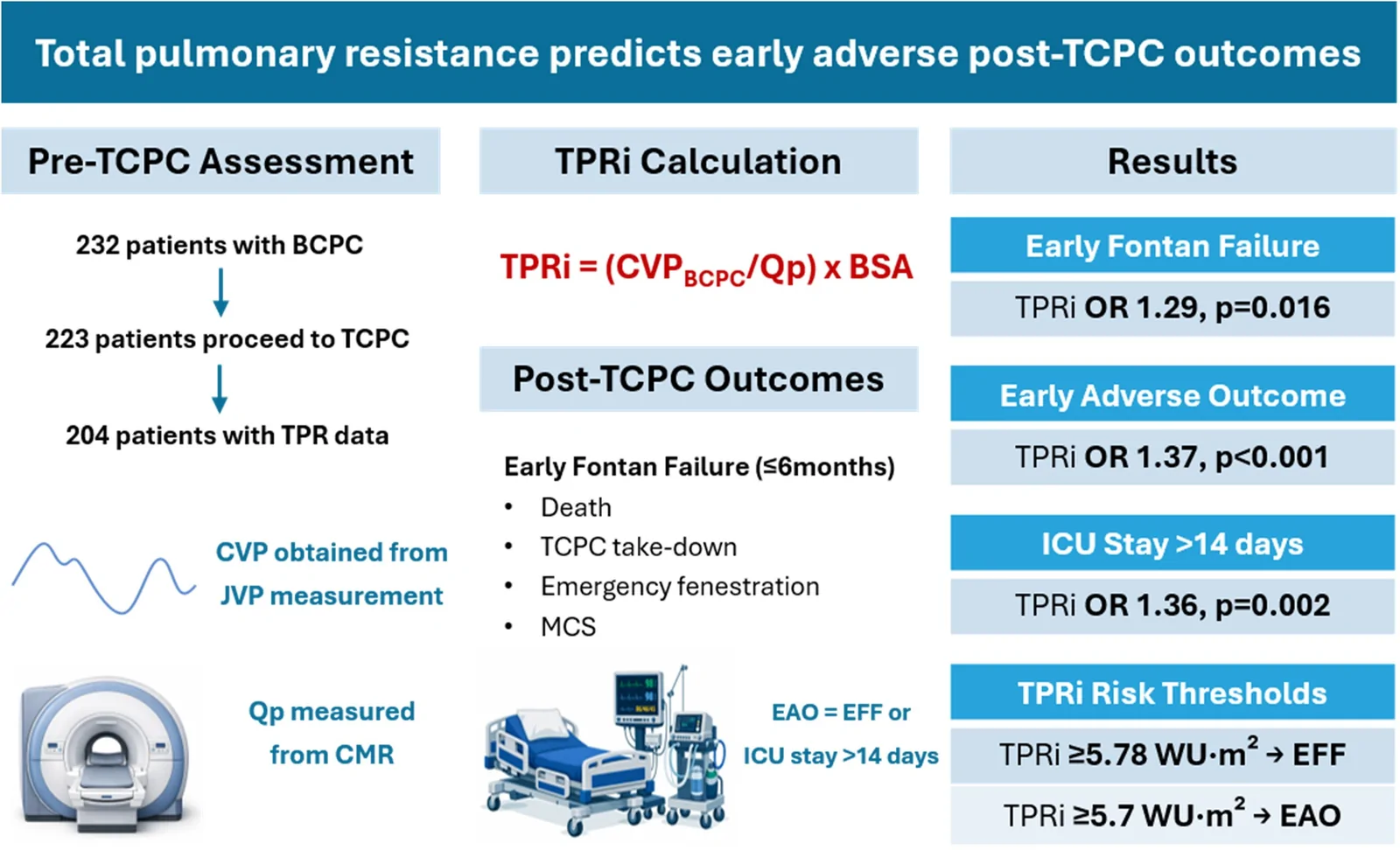
Total pulmonary resistance assessment and association with early outcomes following total cavopulmonary connection.

## Introduction

Patients with single ventricle congenital heart disease undergo a series of staged surgical procedures in early childhood, culminating in a total cavopulmonary connection (TCPC), commonly known as a Fontan circulation. Early Fontan failure (EFF) following TCPC completion remains a serious and potentially fatal complication despite significant advances in surgical technique and peri-operative care. EFF is typically characterized by a low cardiac output state, elevated TCPC circuit pressures, and high volume requirements that are refractory to inotropic support. It remains the primary cause of early mortality following TCPC.^1^ Whilst outcomes have improved with strategies including elective fenestration and avoiding TCPC in those at highest risk of EFF (e.g. single lung Fontan), significant morbidity and variable survival rates persist.^2^ Prolonged intensive care unit (ICU) stay following TCPC can also indicate a suboptimal postoperative course, even in the absence of true EFF. Thus, these outcomes can be combined into a composite measure of early adverse outcome (EAO), providing a broader marker of early adverse TCPC physiology.^3–5^

Accurate prediction of EFF or EAO could inform treatment planning and consent and may be aided by evaluation of pulmonary haemodynamics. The importance of this haemodynamic optimization has been recognized since the 1970s and confirmed in subsequent clinical studies.^6,7^ However, accurately assessing pulmonary haemodynamics in patients with a bidirectional cavopulmonary connection (BCPC) presents substantial technical challenges.

In this study, we propose a minimally invasive approach for assessing pulmonary haemodynamics in patients with a BCPC by combining cardiovascular magnetic resonance (CMR)-derived pulmonary blood flow with direct central venous pressure (CVP) measurement, thereby avoiding cardiac catheterization. Specifically, we measure total pulmonary resistance (TPR), which reflects the combined haemodynamic impedance imposed by pulmonary vascular resistance (PVR), left atrial pressure (LAP), and any significant proximal vascular stenoses.

Our study aimed to (1) estimate TPR at the BCPC stage using data routinely collected during pre-TCPC assessment and (2) determine the association between TPR, EFF (≤6months post-TCPC), post-operative ICU stay >14 days, and EAO (defined as EFF or post-operative ICU stay >14 days).

## Materials and methods

### Study population

**Figure 1:**
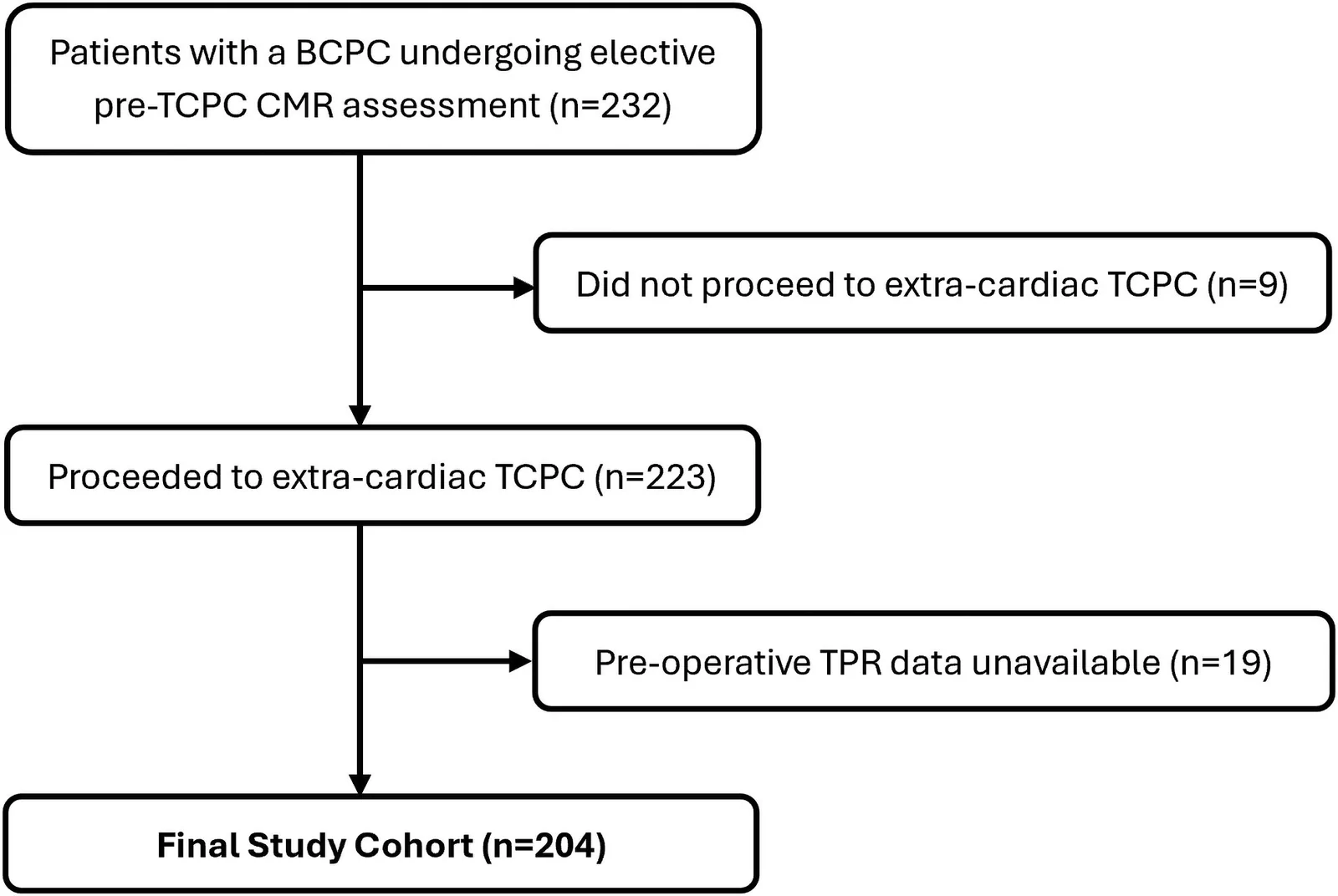
Flow diagram illustrating patient selection and formation of the final analytic cohort.

The study cohort included patients with a BCPC between January 2006 and January 2026 who underwent elective pre-TCPC CMR assessment and subsequently TCPC completion at Great Ormond Street Hospital for Children, London. Of 232 patients with a BCPC, 96% (*n* = 223) proceeded to extra-cardiac TCPC (with or without elective fenestration). Pre-operative TPR data were available for 204 patients, forming the final cohort for all subsequent analyses (*Figure 1*). Patient demographics and clinical details were obtained from medical records. Informed consent for the use of imaging and clinical data was obtained from all parents or guardians of patients included in the study. The study protocol conforms to the ethical guidelines of the 1975 Declaration of Helsinki and was approved by the local committee of the UK national research ethics service (06/Q0508/124).

### CMR imaging

All CMR studies were performed on a 1.5T MR scanner (Avanto; Siemens Medical Systems, Erlangen, Germany) under general anaesthesia, in accordance with our institutional protocol for all pre-TCPC CMR imaging.

#### Flow imaging

Quantitative pulmonary and systemic flows were assessed using retrospectively gated, velocity-encoded phase-contrast CMR. Data were acquired using either a free-breathing Cartesian sequence with three signal averages or a spiral sequence during a 5–8 s breath-hold.^8^ Flow measurements were obtained at the superior vena cava (SVC) near the cavo-pulmonary anastomosis, inferior vena cava (IVC) at diaphragmatic level, pulmonary trunk (where present), proximal branch pulmonary arteries (PAs), proximal pulmonary veins, and ascending aorta. Pulmonary blood flow (*Q*_p_) was derived from SVC flow in patients without residual antegrade pulmonary blood flow and as the sum of SVC flow and native PA flow in patients with persistent antegrade pulmonary blood flow. Vessel segmentation was performed using a semi-automated vessel edge detection algorithm (OsiriX; OsiriX Foundation, Bernex, Switzerland) with manual correction by the operator where required.

#### Quantification of ventricular volumes and function

Ventricular volumes were measured using a retrospectively gated, multi-slice short axis, steady-state free-precession cine sequence.^9^ Individual slices were acquired during 5–10 s breath-holds. End-diastolic and end-systolic volumes of the single functional ventricle were quantified via manual segmentation using an in-house OsiriX plug-in. Stroke volume and ejection fraction were calculated from volumetric data.

#### Anatomical assessment

Arterial and venous anatomy were evaluated using gadolinium-enhanced MR angiography during a 20–30 s breath-hold.^10^ Two consecutive angiograms were performed: the first delineating systemic arterial anatomy and the second capturing second-pass venous and pulmonary arterial anatomy.

### CVP measurement

CVP was measured via ultrasound-guided right internal jugular venous catheterization (Abbocath-T 22G; Venisystems, Lake Forest, Ill) following CMR acquisition during the same general anaesthetic.^11^ Mean CVP (CVP_BCPC_) was measured at passive end-expiration over several respiratory cycles. Measurements were performed in resting, supine patients under the same conditions as the CMR study.

### Deriving TPR

Pressure and flow metrics were integrated to calculate TPR indexed (TPRi) to body surface area (BSA), as follows:


$$\mathrm{TPRi}=(\mathrm{CV}P_{\mathrm{BCPC}}/\mathrm{Qp})\times \mathrm{BSA}$$

where *Q*_p_ was derived from CMR by summing SVC flow and native PA flow (where present) and CVP_BCPC_ was obtained from direct measurement of jugular venous pressure. The unit of TPRi is Wood units·m^2^ (WU·m^2^).

### Outcome measures

The primary outcomes were as follows:

1. EFF, defined as all-cause mortality, TCPC take-down, emergency fenestration, or requirement for mechanical circulatory support (MCS), occurring ≤6 months post-TCPC completion
2. Prolonged post-operative ICU stay (>14 days); and
3. EAO, defined as the composite endpoint of EFF or post-operative ICU stay >14 days

The composite EAO outcome comprised EFF and prolonged ICU stay >14 days. Although these outcomes differ in severity, both represent clinically important manifestations of adverse early adaptation to TCPC physiology. This composite measure was selected to increase statistical power given the low incidence of EFF, while the individual outcomes were also analysed separately.^12^

### Statistics

RStudio (R version 4.4.2; R Foundation for Statistical Computing, Vienna, Austria) was used for statistical analysis and figures. Continuous variables are presented as median (interquartile range) and categorical variables as counts (percentage). The prognostic utility of conventional CMR-derived metrics, including TPR and clinical outcomes, was assessed using logistic regression. Multivariable models including clinically relevant covariates were used to assess independent relationships and *P*-values <0.05 were considered statistically significant. Given the limited number of EFF events, multivariable modelling was restricted to two covariates to minimize the risk of overfitting. Firth penalized logistic regression was performed as a sensitivity analysis to assess the robustness of the multivariable model.

Non-parametric receiver operating characteristic (ROC) analysis was performed to assess the ability of TPRi to discriminate EFF and EAO. The area under the ROC curve (AUC) was calculated using the trapezoidal rule. To prioritize the identification of patients at higher risk of adverse outcomes, the optimal cut-off value was determined by selecting the threshold with the highest specificity among those achieving a minimum sensitivity of 0.75. Sensitivity and specificity were then calculated at the derived threshold.^13^

## Results

### Pre-TCPC haemodynamics and CMR metrics

CMR and CVP data were obtained in 204 patients (male 60.3%, female 39.7%) prior to TCPC completion under general anaesthesia. Median TCPC age was 4 years. 80 patients (39%) had a diagnosis of hypoplastic left heart syndrome and 108 (53%) had a Damus–Kaye–Stansel anastomosis. Patient characteristics for the study cohort are described in *Table 1*.

**Table 1:** Patient characteristics in the final study cohort, *n* = 204

Parameter	Median (IQR) or No. (%)
Male (%)	123 (60.3%)
Age at BCPC (years)	0.5 (0.3–0.8)
Age at CMR (years)	3.0 (2.0–3.0)
Age at TCPC (years)	4.0 (3.0–4.0)
Time from CMR to TCPC (days)	189 (132–346)
BSA at CMR (m^2^)	0.6 (0.6–0.7)
CVP at CMR (mmHg)	12.0 (10–13.3)
Heart rate (beats/minute)	100.0 (90.0–108.0)
Pre-TCPC SpO_2_ (%)	86.0 (82.0–88.0)
Hypoplastic left heart syndrome	80 (39.2%)
Damus–Kaye–Stansel	108 (52.9%)
Isomerism of left or right atrial appendage	8 (3.9%)
Elective fenestration	127 (62.3%)
Indexed total pulmonary resistance (WU·m^2^)	5.7 (4.3–6.8)
Total indexed Qp (L/min/m^2^)	2.2 (1.7–2.6)
Indexed end-diastolic volume (mL/m^2^)	92.2 (80.4–105.6)
Indexed end-systolic volume (mL/m^2^)	38.6 (31.3–48.7)
Indexed stroke volume (mL/m^2^)	52.5 (45.7–59.7)
Ejection fraction (%)	57.0 (52.0–63.0)
Cardiac index (L/min/m^2^)	5.6 (4.8–6.4)
AV valve regurgitant fraction (%)	9.0 (0.0–14.3)
ICU length of stay (days)	3.0 (2.0–6.0)

AV, atrioventricular; BCPC, bidirectional cavopulmonary connection; BSA, body surface area; CMR, cardiovascular magnetic resonance; CVP, central venous pressure; IQR, interquartile range; ICU, intensive care unit; Q_p_, pulmonary blood flow; SpO_2_, oxygen saturation; TCPC, total cavopulmonary connection.

### Early post-TCPC outcomes

Early outcomes including time from TCPC and EFF type are reported in *Table 2*. In summary, 10 patients (4.9%) developed EFF within 6 months of TCPC. Specific EFF types included (i) emergency fenestration in five patients (one of whom later had an emergency TCPC take-down), (ii) emergency TCPC take-down in one patient, (iii) MCS in two patients (both supported with veno-arterial extracorporeal membrane oxygenation, and (iv) death in two patients (death also occurred in two other patients with EFF defined first by emergency intervention: one following emergency fenestration and one following emergency TCPC take-down).

**Table 2:** Early Fontan failure outcome data for patients

Case	Diagnosis	Isomerism	TPRi (WU·m^2^)	Elective fenestration	Emergency fenestration	TCPC take-down	MCS	Early death
1	uAVSD	No	12.1	Yes	No	Yes	No	Yes
2	HLHS	No	5.8	No	Yes	No	No	No
3	HLHS	No	5.8	No	Yes	No	No	No
4	PAt	No	6.5	No	Yes	Yes	No	No
5	PAt, DORV, AVSD	Yes	5.2	No	Yes	No	No	Yes
6	HLHS	No	7.6	Yes	No	No	Yes	No
7	DILV, PS	No	4.4	Yes	Yes	No	No	No
8	uAVSD, DORV	Yes	7.3	Yes	No	No	Yes	No
9	PAt, uAVSD	Yes	11.5	Yes	No	No	No	Yes
10	HLHS	No	11.9	Yes	No	No	No	Yes

AVSD, atrioventricular septal defect; DILV, double inlet left ventricle; DORV, double outlet right ventricle; HLHS, hypoplastic left heart syndrome; MCS, mechanical circulatory support; PAt, pulmonary atresia; PS, pulmonary stenosis; TCPC, total cavopulmonary connection; TPRi, indexed total pulmonary resistance; uAVSD, unbalanced atrioventricular septal defect.

Of the two deaths with no emergency intervention, one patient died from necrotizing pancreatitis during their post-operative hospital stay (Case 9) and one patient died from protein-losing enteropathy (PLE) and possible cardiac arrhythmia, complicated by COVID-19 infection (Case 10).

The median length of ICU stay for all patients was 3 days (IQR 2–6 days). 13 patients (6.4%) had a post-operative ICU stay greater than 14 days. In total, 16 patients (7.8%) met the composite endpoint (EAO), comprising all patients with EFF (*n* = 10) together with those without EFF who required prolonged ICU stay (*n* = 6).

### Predictors of EFF

**Figure 2:**
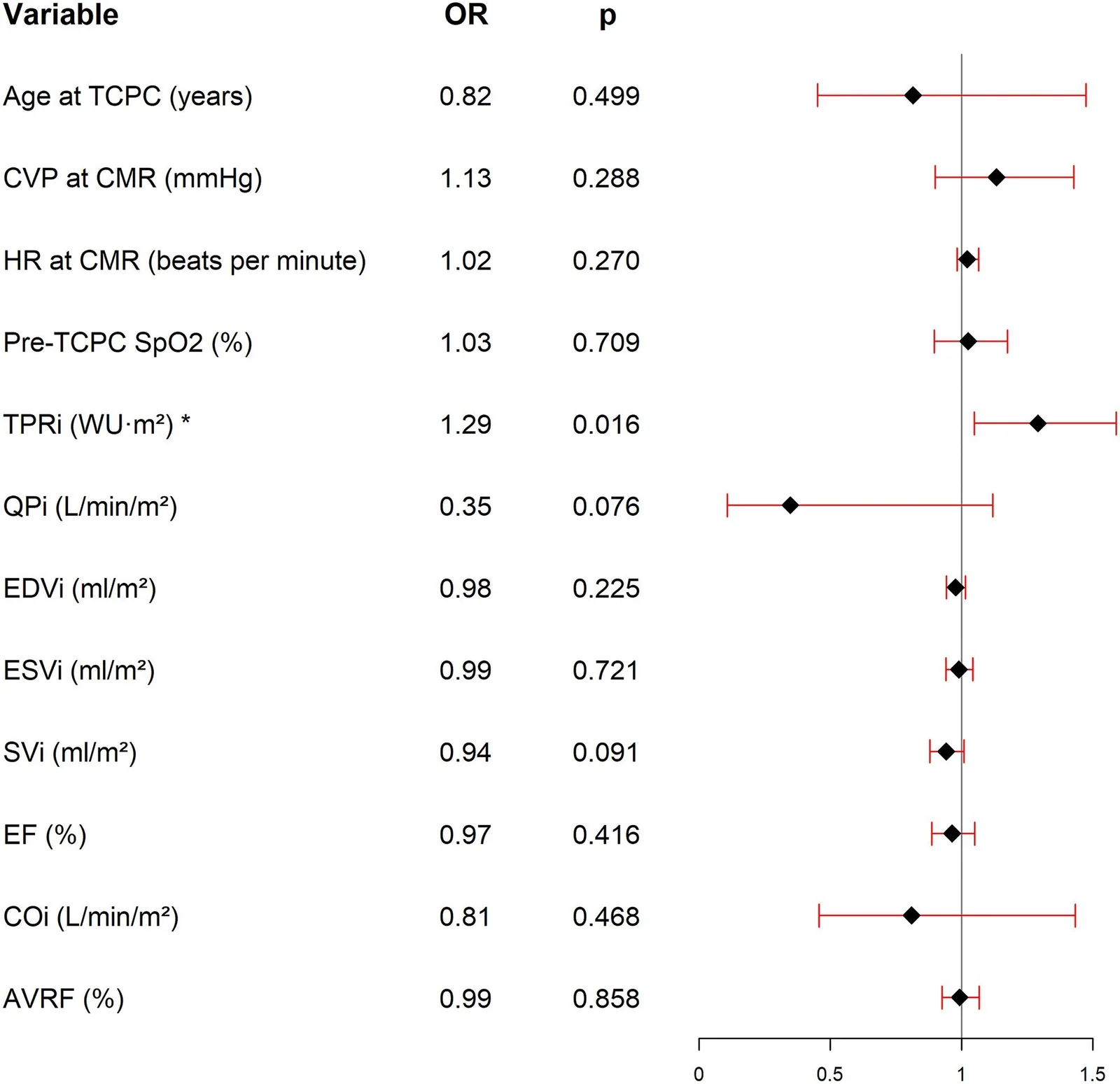
**Forest plot of univariate Odds Ratios for Early Fontan Failure (EFF)**. Points represent odds ratios (ORs) with horizontal lines indicating 95% confidence intervals. ORs and corresponding *P*-values are displayed alongside each variable. Variables marked with an asterisk (*) were significantly associated with EFF (*P* < 0.05). Variables are indexed to BSA, where applicable. AVRF, atrioventricular valve regurgitant fraction; CMR, cardiovascular magnetic resonance; COi, indexed cardiac output; CVP, central venous pressure; EDVi, indexed end-diastolic volume; EF, ejection fraction; ESVi, indexed end-systolic volume; HR, heart rate; Q_p_i, indexed pulmonary blood flow; SpO_2_, oxygen saturation; SVi, indexed stroke volume; TCPC, total cavopulmonary connection; TPRi, indexed total pulmonary resistance.

On univariate analysis, higher TPRi [odds ratio (OR) 1.29, 95% confidence interval (CI) 1.05–1.59, *P* = 0.016] and isomerism (OR 16.20, 95% CI 3.21–81.70, *P* < 0.001) were associated with EFF. No other CMR-derived metrics, clinical variables, or elective fenestration (OR 0.90, 95% CI 0.25–3.31, *P* = 0.880) were associated with EFF (all *P* > 0.05) (*Figure 2*).

**Figure 3:**
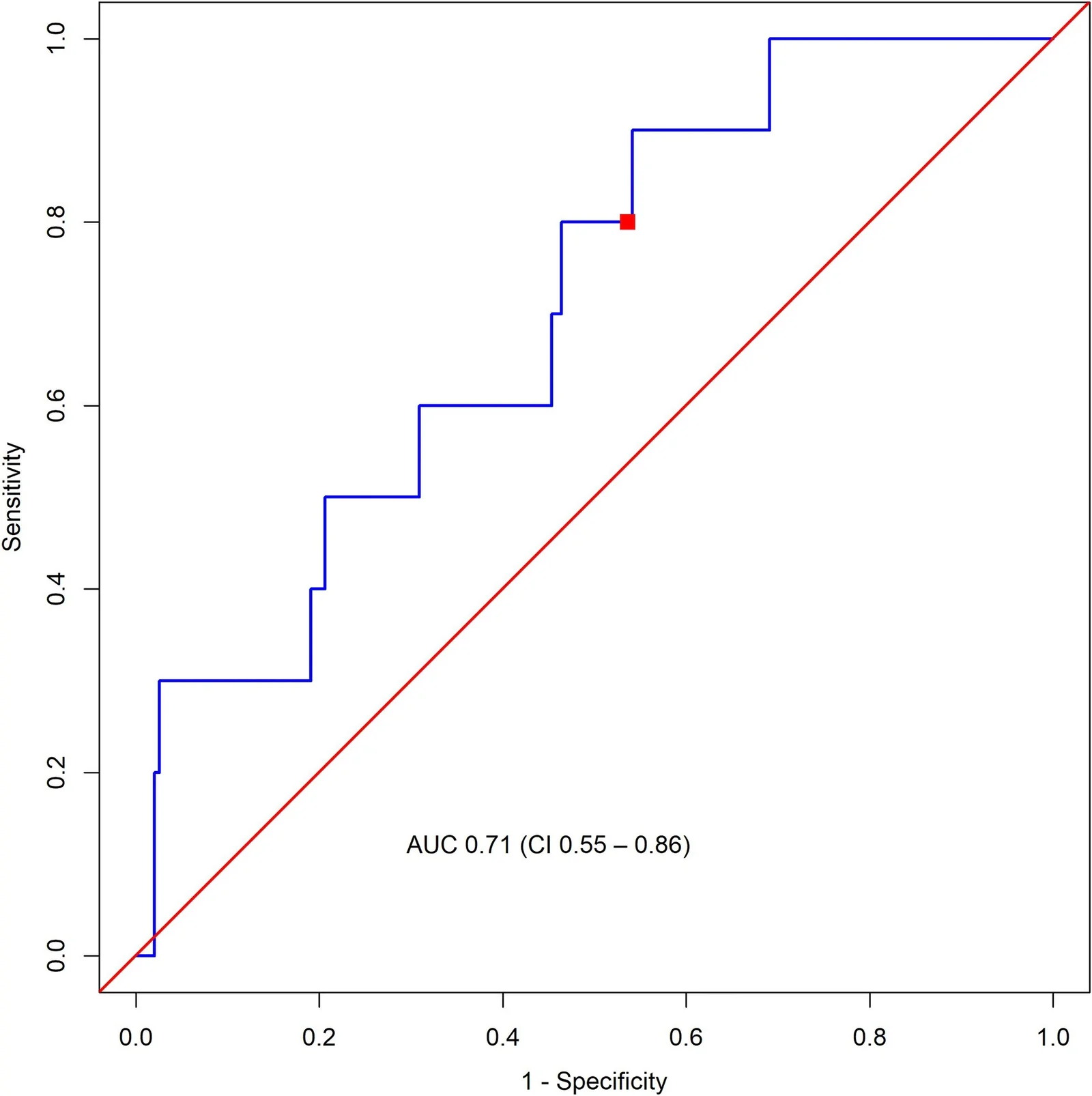
**ROC for indexed total pulmonary resistance (TPRi) and early Fontan failure (EFF)**. AUC 0.71 (95% CI 0.55–0.86, *P* = 0.008), sensitivity of 0.80 and specificity of 0.54 at cut-off point of 5.78 WU·m^2^.

Given the limited number of EFF events (*n* = 10), multivariable modelling was restricted to the two variables associated with EFF on univariate analysis (TPRi and isomerism), resulting in an events-per-variable ratio of 5. In this model, both TPRi and isomerism remained significant predictors of EFF (TPRi: adjusted OR 1.28, 95% CI 1.02–1.60, *P* = 0.030; isomerism: adjusted OR 14.34, 95% CI 2.68–76.81, *P* = 0.002). Sensitivity analysis using Firth penalized logistic regression yielded similar results, with TPRi (OR 1.28, 95% CI 1.02–1.58, *P* = 0.033) and isomerism (OR 13.64, 95% CI 2.61–66.56, *P* = 0.003) remaining significant predictors. Based on ROC analysis, a threshold of 5.78 WU·m^2^ yielded 80% sensitivity and 54% specificity for predicting EFF [AUC 0.71 (95% CI 0.55–0.86, *P* = 0.008)] (*Figure 3*).

### Predictors of prolonged ICU stay

Patients with higher TPRi were more likely to require an ICU stay exceeding 14 days (OR 1.36, 95% CI 1.13–1.65, *P* = 0.002). Reduced *Q*_p_i (OR 0.28, 95% CI 0.10–0.83, *P* = 0.022) and isomerism (OR 20.78, 95% CI 4.46–96.81, *P* < 0.001) were also associated with prolonged ICU admission. No other CMR-derived metrics, clinical variables, or elective fenestration (OR 3.56, 95% CI 0.77–16.49, *P* = 0.105) were associated with this outcome (all *P* > 0.05).

### Predictors of EAO

**Figure 4:**
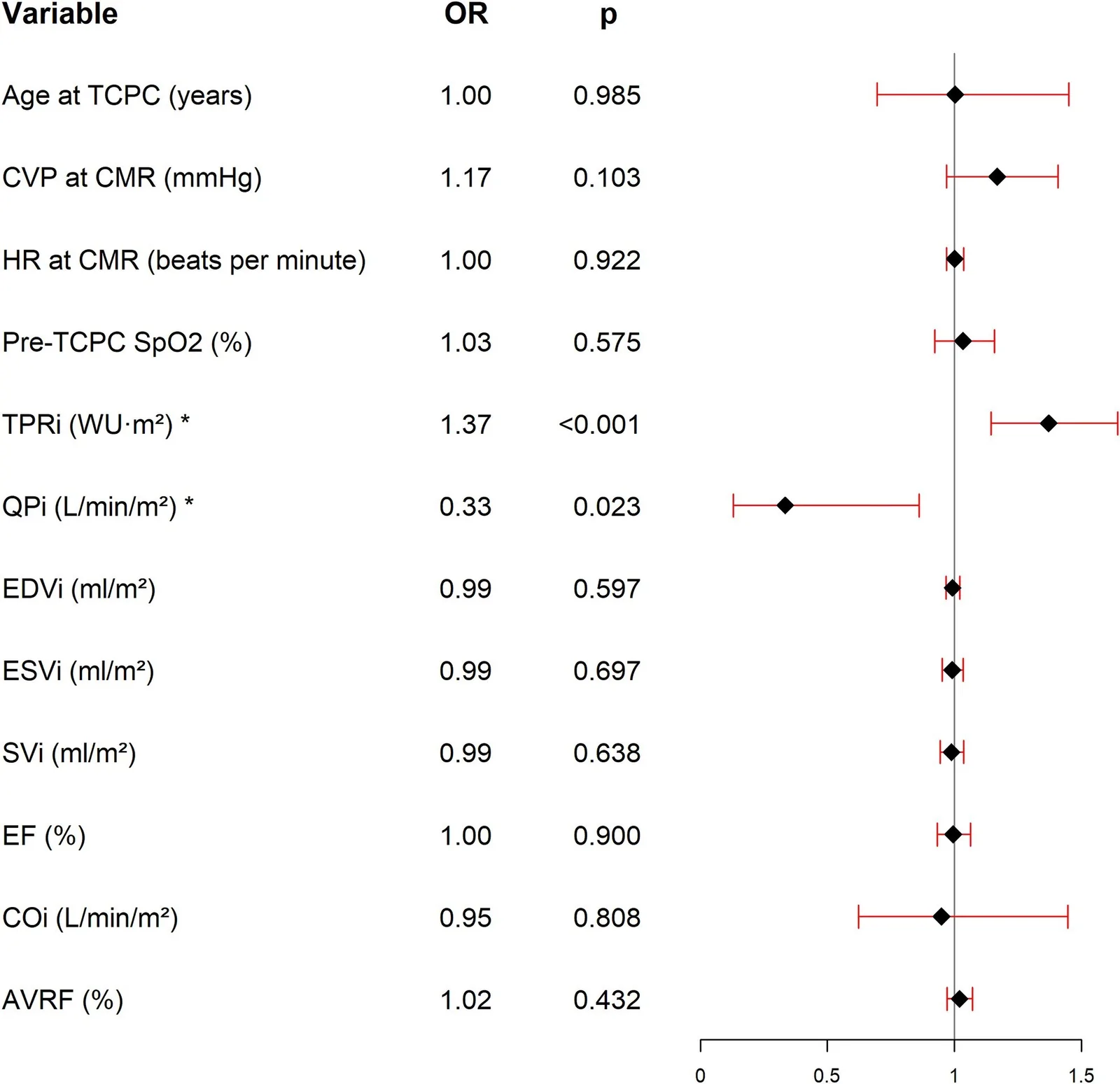
**Forest plot of univariate odds ratios for early adverse outcome (EAO)**. Points represent odds ratios (ORs) with horizontal lines indicating 95% confidence intervals. ORs and corresponding *P*-values are displayed alongside each variable. Variables marked with an asterisk (*) were significantly associated with EAO (*P* < 0.05). Variables are indexed to BSA, where applicable. AVRF, atrioventricular valve regurgitant fraction; CMR, cardiovascular magnetic resonance; COi, indexed cardiac output; CVP, central venous pressure; EDVi, indexed end-diastolic volume; EF, ejection fraction; ESVi, indexed end-systolic volume; HR, heart rate; Q_p_i, indexed pulmonary blood flow; SpO_2_, oxygen saturation; SVi, indexed stroke volume; TCPC, total cavopulmonary connection; TPRi, indexed total pulmonary resistance.

Higher TPRi (OR 1.37, 95% CI 1.14–1.64, *P* < 0.001), lower *Q*_p_i (OR 0.33, 95% CI 0.13–0.86, *P* = 0.023), and isomerism (OR 15.33, 95% CI 3.41–68.99, *P* < 0.001) were associated with EAO on univariate analysis. No other CMR-derived metrics, clinical variables, or elective fenestration (OR 1.90, 95% CI 0.59–6.13, *P* = 0.280) were associated with EAO (all *P* > 0.05) (*Figure 4*).

**Figure 5:**
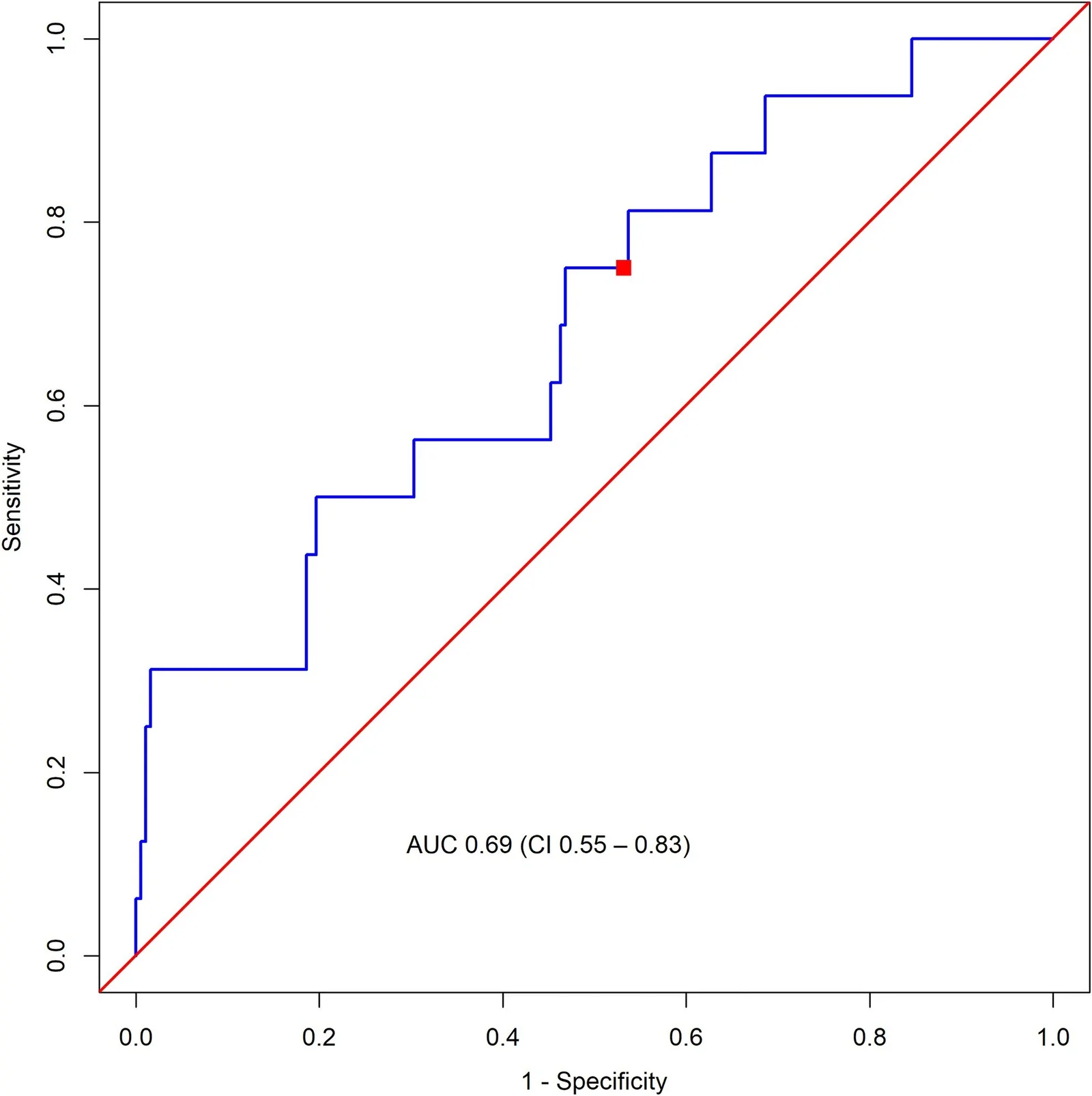
**ROC for indexed total pulmonary resistance (TPRi) and early adverse outcome (EAO)**. AUC 0.69 (95% CI 0.55–0.83, *P* = 0.009), sensitivity of 0.75 and specificity of 0.53 at cut-off point of 5.7 WU·m^2^.

A multivariable model including TPRi and isomerism demonstrated that both variables remained significant predictors of EAO (TPRi: adjusted OR 1.37, 95% CI 1.13–1.66, *P* = 0.001; isomerism: adjusted OR 14.53, 95% CI 2.92–72.31, *P* = 0.001). Sensitivity analysis using Firth penalized logistic regression was consistent with these findings (TPRi: OR 1.36, 95% CI 1.13–1.64, *P* = 0.001; isomerism: OR 13.62, 95% CI 2.89–65.40, *P* = 0.001). A threshold of TPRi 5.7 WU·m^2^ yielded 75% sensitivity and 53% specificity for identifying EAO (AUC 0.69 [95% CI 0.55–0.83, *P* = 0.009]) (*Figure 5*).

## Discussion

In this large single-centre cohort of patients undergoing TCPC, we demonstrated that TPR can be measured at the BCPC stage using data obtained from routine CMR and CVP assessment. Higher TPR was associated with an increased risk of both EFF and EAO, even after adjustment for important clinical covariates such as isomerism. In contrast, clinical characteristics, pre-operative CVP, and CMR-only metrics included in this study did not predict these outcomes. Together, these findings highlight the importance of combining pressure and flow data to achieve a more comprehensive haemodynamic assessment prior to TCPC completion.

Historical studies consistently identified elevated pre-operative CVP or PA pressure as risk factors for EFF.^14–16^ However, we did not observe an association between pre-operative CVP and acute outcomes in our cohort. This discrepancy may partly reflect contemporary patient selection, increased utilization of elective fenestration, and the overall decline in EFF rates from 15–27% in historical series to approximately 1–2% in current practice. Isolated CVP may also be an inherently misleading marker. In patients with elevated pulmonary vascular impedance, off-loading through superior-to-inferior veno-venous collaterals reduces pulmonary blood flow and may produce a falsely reassuring CVP.^17^ Such patients may therefore appear low risk by CVP criteria alone, despite underlying pulmonary vascular abnormalities that are only unmasked after TCPC completion, when lower body and collateral pathways are incorporated into the circuit.

Formal catheterization with calculation of PVR may provide a better method of assessing these patients. However, invasive catheterization is associated with (i) increased risk of vascular injury, arrhythmia, and thromboembolism and (ii) substantial costs from procedural time, staffing, consumables, and post-procedural monitoring. More importantly, catheterization relies on Fick-based calculations for flow quantification, which require assumed oxygen consumption values that are prone to significant error in paediatric patients and those with complex circulations. For this reason, PVR measurements have not consistently been shown to predict EFF.^18,19^

An alternative approach is CMR, which provides excellent anatomical and functional assessment, including accurate quantification of ventricular volumes, valvular regurgitation, systemic and pulmonary blood flow, and vascular anatomy including collaterals. These data have prognostic value for several important clinical outcomes including post-operative pleural drainage and hospital stay.^20,21^ However, CMR cannot directly measure intravascular pressures. Whilst some studies have explored CMR-derived surrogates for pressure estimation, these remain investigational and lack the precision required for reliable resistance calculations. Our findings reinforce this limitation: CMR-derived metrics alone, including ventricular volumes, ejection fraction, and indexed pulmonary blood flow, were not independently associated with EFF. This underscores that flow data, whilst necessary, is insufficient without corresponding pressure measurements to characterize the haemodynamic burden imposed by the pulmonary vascular bed. Hybrid CMR–catheter techniques have been described for accurate PVR assessment; however, they are costly and require specialist equipment and expertise.^22–24^

The approach used in this study addresses these limitations by pairing accurate CMR flow quantification with direct pressure measurement, without requiring full cardiac catheterization. CVP measurement via jugular venous access is technically straightforward, is of low risk, and can be performed under the same anaesthetic as CMR, avoiding additional procedures. This yields a physiologically complete measure of total pulmonary impedance that reflects the total opposition to pulmonary blood flow, incorporating contributions from PVR, LAP, and any proximal vascular stenoses. It should be noted that TPR differs mathematically from PVR with the following relationship: TPRi = PVRi + LAP/*Q*_p_i. Based on the assumptions outlined by Suthar et al., in a typical patient with LAP of 7.8 mmHg and a BCPC *Q*_p_i of 2.2 L/min/m^2^, a threshold TPRi of 5.7 WU·m^2^ would equate to a PVRi of 2.2 WU·m^2^.^25^

Importantly, TPR should not be regarded as interchangeable with PVR. As a composite haemodynamic measure, an elevated TPR can arise through different mechanisms, so its clinical significance may vary between patients. Whilst this heterogeneity limits direct comparison with catheter-derived PVR, it may also be a strength, as TPR reflects the overall impedance of the TCPC circuit. When assessing TCPC suitability, an elevated TPR is therefore best regarded as a marker of an unfavourable pulmonary circulation, regardless of the underlying mechanism, and may be used to prompt further clinical investigation.

From a health-economic perspective, avoiding diagnostic catheterization reduces procedural costs, shortens hospital episodes, and minimizes cumulative anaesthetic and ionizing radiation exposure in a population that will require lifelong surveillance. Whilst formal cost-effectiveness analysis was beyond the scope of this study, the potential for a safer, less invasive, and more accessible approach to pre-TCPC risk stratification warrants further evaluation. Importantly, this approach is generalizable to centres performing routine pre-TCPC CMR, even where interventional MRI catheterization facilities are unavailable.^22^

### EFF and adverse outcomes

In this study we have used a robust definition of EFF, characterized by verifiable severe clinical events including, death, TCPC take-down, emergency fenestration, and requirement for MCS. We also incorporated ICU stay >14 days into our composite EAO to capture a broader spectrum of clinically significant early morbidity following TCPC.^4,26^ In this study, we demonstrated that TPR was independently associated with both EFF and EAO, with the only other significant covariate being isomerism/heterotaxy.^24,27^

Isomerism has consistently emerged as one of the most significant pre-operative risk factors for adverse outcomes following TCPC and is re-confirmed in this contemporary series.^14,24,28–30^ The reasons for poorer outcomes independent of pulmonary vascular impendence are multifactorial. Patients with isomerism typically have more complex single ventricle anatomy and may require concomitant intervention at the time of TCPC, entailing a more complicated post-operative course.^31^

Although associated with EFF and EAO, clinical utility is better evaluated through prediction. TPRi demonstrated moderate discriminatory ability for predicting early outcomes following TCPC. A threshold ≥5.78 WU·m^2^ identified EFF with 80% sensitivity and 54% specificity, while ≥5.7 WU·m^2^ identified the composite endpoint of EAO with 75% sensitivity and 53% specificity. These thresholds provide a pragmatic framework for risk stratification but should be interpreted within the broader clinical context. The high sensitivity for EFF/EAO supports the potential role of TPRi as a pre-operative screening tool for early haemodynamic instability, although the modest specificity may result in over-classification of some patients as high risk. Consequently, elevated TPRi should be considered an adjunctive risk marker rather than a standalone determinant of management strategy. At these thresholds, the positive predictive value was low (8.2% for EFF and 11.8% for EAO at the observed event rates of 4.9% and 7.8%, respectively), whereas the negative predictive value was high (98.1% and 96.1%, respectively), indicating that an elevated TPRi modestly raises the index of suspicion while a value below the threshold provides strong reassurance. TPRi may support clinical decision making before TCPC and guide anticipatory care for high-risk cases, e.g. pre-operative optimization, elective fenestration, and availability of MCS. External validation of these thresholds in independent cohorts will be required prior to clinical implementation.

### Limitations

This study has several limitations. First, this is a single-centre retrospective analysis, which may limit generalizability given variation in pre-operative TCPC assessment protocols, surgical techniques, and fenestration strategies across institutions. The proposed approach also relies on routine pre-TCPC CMR and CVP measurement being performed under the same general anaesthetic, practices that may not be routinely available in all centres. Implementation may therefore be less feasible in institutions without established pre-TCPC CMR protocols. However, the retrospective design ensured that TPR was not incorporated into clinical decision-making and therefore could not have influenced outcomes. As such, the observed associations likely reflect true physiological relationships.

Second, the number of EFF and EAO events was small, consistent with contemporary surgical outcomes but limiting statistical power for multivariable modelling. The wide CIs for our effect estimates, particularly for isomerism, reflect this constraint. Larger multi-centre studies are needed to validate these findings and enable more robust adjustment for potential confounders.^32,33^

Third, our definition of EFF encompassed death, TCPC take-down, emergency fenestration, or requirement for MCS within 6 months. Whilst clinically meaningful, this approach may obscure differing risk profiles for each component. The small event numbers precluded separate analysis of individual endpoints. Similarly, EAO combined EFF with prolonged ICU stay (>14 days), integrating outcomes of differing clinical severity. ICU length of stay may also be influenced by institutional practices, peri-operative management strategies, and non-cardiac complications, with varying cut-offs for ‘prolonged stay.’

Fourth, TPR reflects total pulmonary impedance but does not distinguish which of its constituent components, PVR, LAP, or proximal vascular stenoses, is responsible in an individual patient.

Finally, our study focused on early post-TCPC outcomes. The relationship between pre-operative TPR and late TCPC failure, including PLE, plastic bronchitis, and cirrhosis, remains unknown and warrants longitudinal investigation.

## Conclusions

In this large single-centre cohort, we demonstrate that TPR can be calculated at the BCPC stage by integrating routine CMR flow data with direct CVP measurement, avoiding the need for full cardiac catheterization. Elevated TPR is independently associated with EAOs, whereas pressure or flow metrics alone lacked prognostic utility. Early identification of patients at elevated haemodynamic risk may inform strategies such as optimized timing of TCPC, elective fenestration, and anticipatory post-operative care. Incorporating minimally invasive TPR assessment into routine pre-TCPC evaluation has the potential to improve risk stratification.

## Data Availability

The datasets generated and analysed during this study are available from the corresponding author on reasonable request, subject to institutional data governance requirements.

## Lead author biography

Dr Niromila Nadarajan is a Cardiology Higher Specialty Trainee, Clinical Research Fellow and PhD researcher at the Institute of Cardiovascular Science, University College London, UK. Dr Nadarajan’s clinical and research interests are centred around congenital heart disease, with a specialist interest in single ventricle congenital heart disease and improving outcomes in patients with Fontan circulations. Her current research focuses on Fontan-associated liver disease, aiming to advance understanding of its pathophysiology through interdisciplinary work spanning congenital cardiology, hepatology, and imaging.
